# Irreducible inferior shoulder dislocation requiring open reduction: A case report

**DOI:** 10.1016/j.tcr.2021.100426

**Published:** 2021-02-10

**Authors:** Taichi Nishimura, Ryogo Furuhata, Kentaro Okuma, Toshiki Iyanagi, Yusaku Kamata, Hideo Morioka

**Affiliations:** Department of Orthopaedic Surgery, National Hospital Organization Tokyo Medical Center, Tokyo, Japan

**Keywords:** Inferior shoulder dislocation, Rotator cuff tear, Irreducible, Open reduction, Greater tuberosity fracture, Case report

## Abstract

In inferior shoulder dislocation (ISD) cases, closed reduction usually achieves reduction and irreducible ISD is extremely rare. To date, only two cases requiring open reduction have been reported. Herein, we describe a case of an irreducible ISD that required open reduction. A 90-year-old woman fell at home and presented to our hospital. Plain radiography revealed a right ISD and greater tuberosity avulsion fracture. Because reduction under general anesthesia was difficult, we performed open reduction. The humeral head was entrapped by the inferior shoulder capsule. Since inferior instability remained after reduction, we reduced and fixed the greater tuberosity fracture and repaired the rotator cuff tear (RCT). This case suggested that humeral head entrapment by the inferior capsule and decreased force couple toward the humeral head by the greater tuberosity fracture and RCT cause irreducibility. Moreover, since instability can remain after reduction for ISD accompanying greater tuberosity fracture or RCT, preparing for implantations to repair these lesions is recommended.

## Background

Irreducible inferior shoulder dislocation (ISD) occurs rarely (0.5% of all shoulder dislocations), and the affected shoulder is characterized by a fixed abducted position [1]. Reduction procedures for ISD include the traction-countertraction method and the two-step maneuver procedures [2,3]. In the traction-countertraction method, the upper limb is pulled while a counter traction is applied from above the acromion, and the shoulder joint is adducted [2]. In the two-step maneuver process, inferior dislocation is converted to anterior dislocation and the upper limb is then pulled and rotated [3]. Although these closed reduction procedures were effective to achieve reduction previously [2,3], for the rarely occurring irreducible ISD, only two cases requiring open reduction have been reported until now [4,5]. Moreover, the factors that affect closed reduction failure for ISD are not fully understood.

Herein, we report a case of irreducible ISD that required open reduction. This case demonstrated that irreducibility was attributable to the button-hole entrapment of the humeral head by the inferior capsule and the failure of the force couple to reach toward the humeral head by the rotator cuff tendon due to concurrent greater tuberosity fracture and rotator cuff tear (RCT).

## Case presentation

**Fig. 1:**
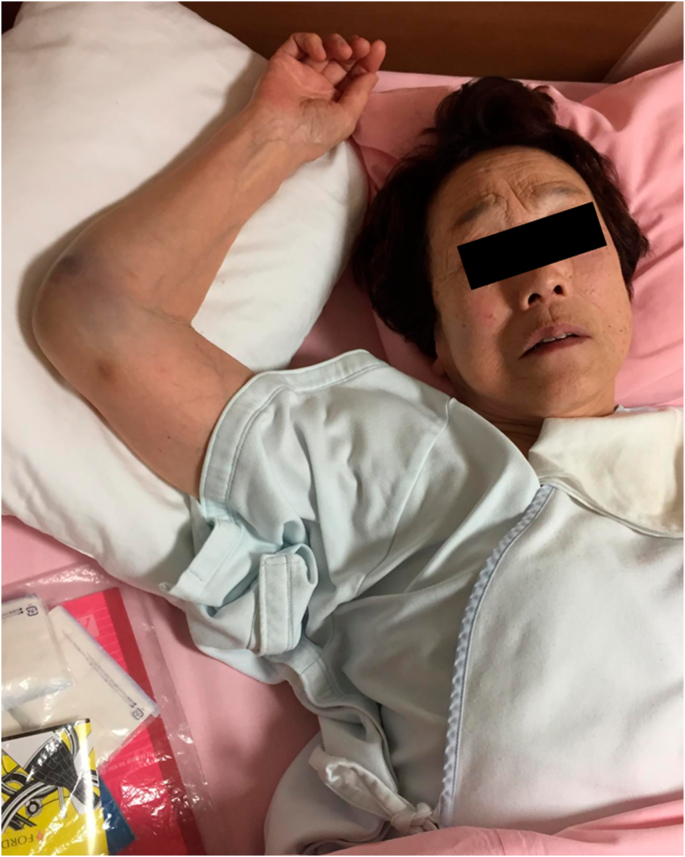
Appearance during shoulder dislocation. The right shoulder joint was locked in 120° of abduction, and moving the shoulder was difficult.

**Fig. 2:**
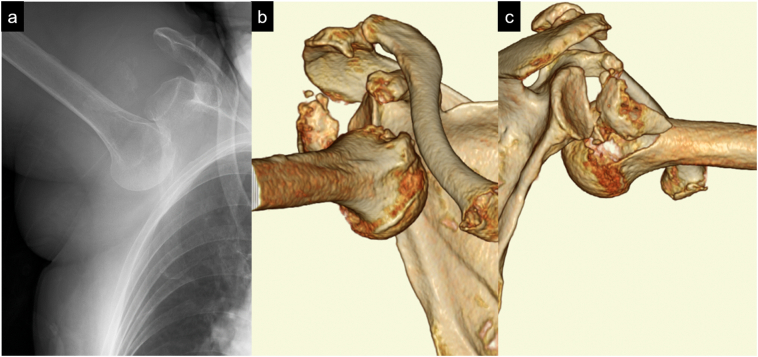
Plain radiograph and computed tomography of the right shoulder. (a) Initial radiographs showing right shoulder inferior dislocation. (b and c) Computed tomography performed during shoulder dislocation revealed the avulsion fracture of the greater tuberosity.

A 90-year-old woman fell at home and presented to our hospital. She had a history of hypertension. The right shoulder joint was locked in 120° of abduction; thus, moving the shoulder was difficult (Fig. 1). No findings suggestive of nerve or vascular injury were observed. Plain radiography showed an inferior dislocation fracture of the right shoulder joint (Fig. 2a), and computed tomography revealed a right greater tuberosity avulsion fracture, with the greater tuberosity bone defect as the dislocation pathway (Fig. 2b and c). After intra-articular injecting xylocaine in the emergency room, we attempted reduction; however, it was difficult. Therefore, reduction was performed under general anesthesia. We attempted reduction using the traction-countertraction, and in the two-step method under general anesthesia, reduction could not be achieved [2,3]. Thus, we scheduled open reduction with greater tuberosity fracture fixation.

**Fig. 3:**
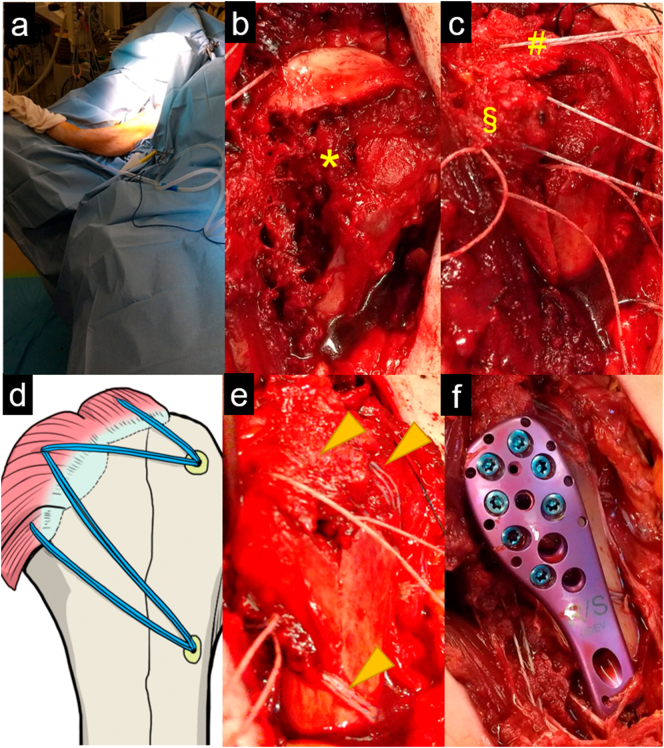
Intraoperative findings of the right shoulder. Since dislocation was difficult under general anesthesia, the patient was placed on a beach chair position with the affected upper limb fixed in abduction during surgery (a). The entrapment of the humeral head by the inferior capsule was removed, and dislocation reduction was conducted. Bone defects were observed at the infraspinatus tendon and teres minor tendon attachment in the greater tuberosity (b). The supraspinatus tendon was completely torn and was sutured and pulled by FiberWire® (#). The infraspinatus tendon and teres minor tendon were sutured, and reduction of the greater tuberosity bone fragments (§) was performed (c). The completely torn supraspinatus tendon (**) was sutured via the suture bridge method (arrowhead). The infraspinatus tendon, teres minor tendon, and greater tuberosity bone fragments were reduced and fixed together by suture anchors (arrowhead) (d and e). The temporarily fixed greater tuberosity bone fragment was further fixed by a plate (f).

**Fig. 4:**
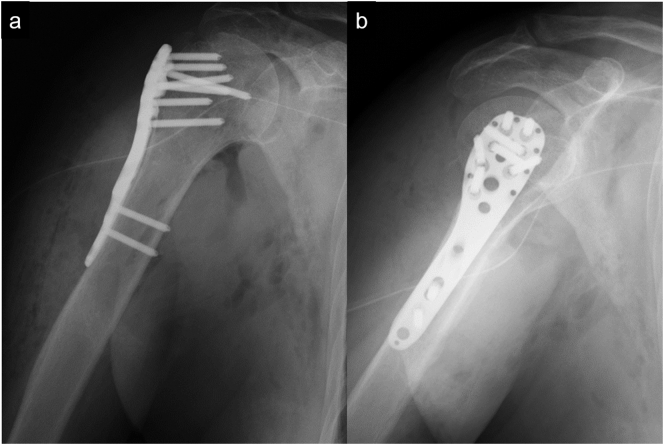
On postoperative plain radiographs, the glenohumeral joint was reduced, and the greater tuberosity was also fixed.

Surgery was performed with the right upper limb fixed in abduction while the patient remained in a beach chair position (Fig. 3a). Through the deltoid-pectoral approach, a button-hole humeral head entrapment by the inferior capsule was identified. After the entrapment was removed, dislocation was reduced using the two-step maneuver. However, dislocation easily recurred when the shoulder was elevated to 90° or more. Bone defects were observed at the infraspinatus tendon and teres minor tendon attachment in the greater tuberosity (Fig. 3b). Additionally, we identified the complete tear of the supraspinatus tendon. We considered that the concurrent greater tuberosity fracture and RCT caused instability; therefore, we repaired these lesion. The greater tuberosity bone fragment was reduced by using FiberWire® (Arthrex, Naples, FL, USA) in the infraspinatus and teres minor tendons. The completely torn supraspinatus tendon was also repaired using FiberWire® (Fig. 3c). A JuggerKnot® anchor (Zimmer Biomet, Warsaw, IN, USA) was inserted medially into the greater tuberosity, and another Quattro® Link Knotless Anchor (Zimmer Biomet, Warsaw, IN, USA) was inserted laterally into the greater tuberosity while retaining the reduction position (Fig. 3c). We temporarily fixed the greater tuberosity and repaired the supraspinatus tendon using the suture bridge technique (Fig. 3d and e). Subsequently, we fixed the greater tuberosity fracture using the MODE Proximal Humeral Plate® (MDM, Tokyo, Japan) (Fig. 3f). Postoperative plain radiographs are shown in Fig. 4.

Postoperatively, the patient was immobilized in an abduction brace for 6 weeks. Passive range of motion exercises were started at 3 days postoperatively, and active range of motion exercises were started at 6 weeks postoperatively. No recurrent dislocation or instability was noted at 10 months postoperatively.

## Discussion and conclusions

The present case highlighted two clinical issues: First, the irreducibility of ISD could be attributed to the button-hole entrapment of the humeral head by the inferior capsule and the decreased stabilization of the humeral head by the rotator cuff tendon (due to the RCT and the greater tuberosity fracture occurring concurrently). Most ISDs can be reduced successfully via the traction-countertraction or the two-step maneuver methods [2,3]. In this case, we attempted both of these procedures under general anesthesia, but they were ineffective. To date, only two case reports have presented irreducible ISD details [4,5]. Frank et al. reported that irreducibility occurred because of direct inhibition from the aberrant (anterior) position of the axillary nerve [4]. Khedr et al. stated that humeral head entrapment in a button-hole through the inferior shoulder joint capsule inhibited reduction [5]. In our case, intraoperative findings revealed that the button-hole entrapment of the humeral head by the inferior capsule caused irreducibility, while the interposition of the axillary nerve was not observed. Additionally, in our case, the disruption of the force couple toward the humeral head by the rotator cuff tendon could inhibit the reduction. Regarding anterior shoulder dislocation, a massive RCT in older individuals and greater tuberosity fracture were thought to be challenging factors for reducing shoulder dislocations [6]. In our case, in addition to the complete rupture of the supraspinatus tendon, the infraspinatus tendon and teres minor tendon were detached from the humeral head. Since a massive RCT or greater tuberosity fracture was also involved concurrently in those reported cases requiring open reductions [4,5], the failure of the forced couple caused by such a RCT or greater tuberosity fracture can be ascertained as one of the factors contributing to the irreducibility of ISD.

Second, instability can remain after reduction for ISD accompanying a greater tuberosity fracture or RCT, as with this patient. Therefore, remaining prepared for possible implantations to repair these lesions following reduction is recommended. In ISDs, the recurrent dislocation rate has not been fully clarified. Some studies have reported satisfactory results with early reduction and 3-week Velpeau fixation and the extremely rare recurrence of inferior dislocation [7]. However, other studies have reported that 8/14 patients with inferior dislocation experienced re-dislocation, with 7 patients requiring surgery [8]. To date, risk factors for inferior shoulder instability after initial reduction has been reported to include concurrent RCTs or bone defects due to concurrent greater tuberosity fractures [9]. In particular, greater tuberosity bone defect of ISD is considered to adversely affect the instability after reduction by a mechanism similar to a Hill-Sachs lesion for recurrent anterior dislocation [9]. In this case, the risk for the instability increased after initial reduction because RCT and greater tubercle fracture were present. The intraoperative findings of our case further suggested that inferior instability remained only after reduction. Previous reports of surgical treatment for recurrent inferior shoulder instability demonstrated favorable results when RCTs, capsulolabral injury, superior labral tear from anterior to posterior, and greater tuberosity fractures associated with ISD were repaired [9]. Thus, repairing such concurrent lesions to restore the force couple would be preferable for ISD complicated with rotator cuff tear and greater tuberosity fractures, as was the case for our patient.

In summary, this case report describes the surgical treatment of irreducible ISD, which occurred in an elderly patient. This case highlights that the button-hole humeral head entrapment by the inferior shoulder capsule and the decreased force couple to the humeral head due to concomitant greater tuberosity fracture or RCT can cause irreducibility, which warrants open reduction. Moreover, since instability can remain after reduction for ISD accompanying a greater tuberosity fracture or RCT, remaining prepared for possible implantations to repair these lesions is recommended.

## Ethics approval and consent to participate

Consent to participate is not applicable in this type of study. A statement of the ethics committee was not required from this anonymized case report in accordance with the legislation of the Institutional Review Committee of our institution.

## Consent for publication

Written informed consent was obtained from the patient for publication of this case report and any accompanying images. A copy of the written consent is available for review by the Editor-in-Chief of this journal.

## CRediT author statement

**Taichi Nishimura**: Data curation, Writing-Original draft preparation, Software. **Ryogo Furuhata**: Writing-Original draft preparation, Conceptualization, Methodology. **Kentaro Okuma**: Data curation, Visualization. **Toshiki Iyanagi**: Investigation. **Yusaku Kamata**: Supervision, Validation. **Hideo Morioka**: Writing- Reviewing and Editing,

## Availability of data and materials

Data that support the findings of this study are available from the corresponding author on reasonable request.

## Funding

The authors certify that they or their institutions did not receive any support (e.g. grants, funding, payment or other benefits) or a commitment or agreement to provide such benefits in connection with the research or preparation of this manuscript, except as disclosed on a separate attachment.

## Declaration of competing interest

None.
